# Moon Sensor Station to Improve the Performance of Lunar Satellite Navigation Systems

**DOI:** 10.3390/s25123675

**Published:** 2025-06-12

**Authors:** Mauro Leonardi, Gheorghe Sirbu, Mattia Carosi, Cosimo Stallo, Carmine Di Lauro

**Affiliations:** 1 Department of Electronic Engineering, Tor Vergata University, Via del Politecnico 1, 00133 Rome, Italy; 2 THALES Alenia Space Italia, Via Saccomuro, 24, 00131 Rome, Italy

**Keywords:** lunar navigation, lunar beacon, differential positioning, pseudolite, ELFO

## Abstract

Today, Moon exploration is driven by the desire to expand the human presence beyond Earth and to use its resources. This requires the development of reliable navigation systems that can provide positioning information accurately and continuously on the lunar surface and orbits. Initiatives such as Moonlight (by ESA) and the Cislunar Autonomous Positioning System project (by NASA) are underway to address this challenge. The aim is to use ranging signals transmitted by satellites, similar to Earth’s GNSS, for lunar user positioning. This paper proposes a solution that involves local sensors deployed on the Moon surface to enhance the performance of the satellite system. These sensors can serve as differential reference stations, correcting satellite pseudorange measurements obtained by lunar surface receivers. The local sensor can also be used as a pseudolite, transmitting satellite-like signals to improve system availability and accuracy in obstructed areas. Additionally, the local sensor can act as an independent beacon that provides range and angle measurements. Higher navigation performance can be achieved by increasing the complexity of the system, depending on the implemented solution. This paper proposes and shows the concept, the intial design, and a preliminary definition of the protocol for the third solution. The three different solutions are compared in terms of position accuracy by exploiting the Cramér–Rao Lower-Bound formulation and Monte Carlo simulations. Finally, possible implementations for future use on the Moon are discussed.

## 1. Introduction

Nowadays, there is a renewed interest in lunar exploration and colonization, driven by the desire to establish a human presence beyond Earth and to exploit the resources available on the Moon [1]. However, the success of such missions is contingent on the development of reliable navigation systems that can provide accurate and continuous positioning information on the lunar surface. Current Earth-based tracking stations, such as the NASA Deep Space Network—DSN [2]—have several limitations in supporting a large number of users, in accuracy, and in availability, e.g., the Moon south pole and the far side exhibit limited or no visibility from Earth-based stations, system capacity, etc. [3,4].

In this regard, the European Space Agency (ESA) is developing the Moonlight initiative, which will use satellite ranging signals to determine the position and velocity of Moon users, similar to the Global Navigation Satellite System (GNSS) [5,6], NASA is also working on the Cislunar Autonomous Positioning System (CAPS) project, with similar aims [7]. Moreover, the Japan Aerospace Exploration Agency (JAXA) has recently launched a research project to explore potential satellite systems for lunar navigation [8], Roscosmos has revealed a plan to establish a lunar satellite navigation system in the time frame from 2036 to 2040 [9]. Finally, China has recently disclosed plans through its space agency, the China National Space Administration (CNSA), to develop a satellite constellation orbiting the Moon, aimed at offering communication and navigation services [10].

Extensive research has been conducted on assessing satellite-based navigation systems for the Moon, focusing on exploiting various orbits to achieve optimal performance. Different investigations, such as in [11,12,13,14,15,16], have highlighted two types of constellations as the best choices for lunar applications, the Elliptical Lunar Frozen Orbits (ELFOs) and the Halo orbits.

Recent advances in satellite coverage analysis, as demonstrated in [17] along with complementary research on elliptical-orbit satellite coverage and agile satellite visibility prediction [18,19], highlight significant steps toward optimizing lunar navigation systems. These works collectively contribute to a more refined understanding of satellite coverage dynamics, addressing both orbital perturbations and geometric considerations, vital to ensure comprehensive coverage and reliable navigation on the Moon.

The most popular solutions [6,14,20] adopt the same approach used in terrestrial satellite navigation systems, i.e., the measurement of at least four pseudoranges from synchronized orbiting satellites. Many evaluations have been conducted on this and other working principles in recent years [12,14,16].

Last but not least, the initial phase of lunar exploration has planned the deployment of a limited number of satellites in lunar orbit [21]. For this reason, the accuracy and availability of the navigation system may not always meet the mission requirements.

In particular, as shown in the previously cited work, in the initial phase and with a reduced number of satellites, the accuracy and availability obtained are limited and do not match the requirements for more demanding scenarios, such as the landing and rover phases of a typical Moon mission.

**Figure 1:**
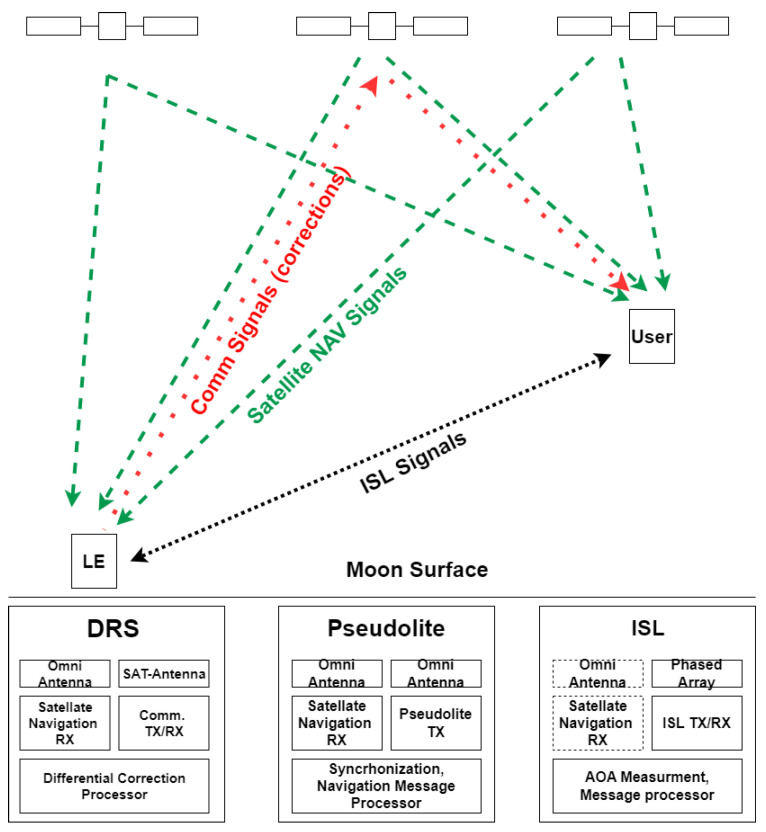
Local sensor working principle scheme.

For this reason, this paper proposes the use of local sensors to improve user positioning performance and compares three configurations with different working methods and complexities. A local sensor can be deployed at a fixed location on the lunar surface and can be used to provide different types of additional information. Three different techniques are proposed, discussed, and evaluated in the following (see Figure 1):

- Differential Reference Station (DRS): the local sensor provides corrections of the satellite pseudorange measurements to the user on the lunar surface;
- Pseudolite (PSL): the local sensor transmits the same signal transmitted by satellites to improve the number of ranging measurements available on the Moon—this solution is similar to the classical GNSS pseudolite;
- Independent Source of Localization (ISL): The local sensor measures range and angles, improving navigation performance.

In this paper, these three possible approaches are evaluated and compared for a typical Moon scenario, near the south pole.

The first solution was originally proposed and evaluated by the authors in [13], via numerical simulations, computing the UERE budget and the DOP information. Moreover, in [22], Akiyama et al. proposed to incorporate pseudorange corrections from a reference station on the Moon’s surface into the navigation message, eliminating the need for additional receiver hardware and achieving a target accuracy of 40 m horizontally, even when users are up to 10 degrees latitude away from the reference station. Finally, the differential approach was also modeled by the authors in [23], obtaining good results up to 25 degrees of latitude away from the reference station, ensuring reliable performance for distant users.

Unfortunately, this approach does not match the more demanding requirements, both in terms of accuracy and availability. The last cited work will be considered as the reference solution in this paper, which introduces another working principle for the local sensor, giving the following new contributions:

- Complete definitions and theoretical models are derived for the ISL;
- A system design of the ISL is created, defining the local sensor’s main building blocks, tasks, and signals, up to the preliminary design of the communication protocols that should be selected;
- A Cramér–Rao Lower Bound (CRLB)-based evaluation method is derived and the three possible working principles are compared with CRLB and Monte Carlo runs;
- Finally, suggestions are derived for the combined use of the three proposed approaches.

The paper is structured as follows: Section 2 describes the one-way navigation techniques for the lunar scenario, the methods used for the evaluation of the navigation performance, and the errors affecting the system; Section 3 describes the local sensor functionalities and models to evaluate its performance as DRS; Section 4 describes the local sensor as pseudolite; Section 5 describes the local sensor as independent beacon; finally, Section 6 reports the results of the simulations, including the description of the constellation considered, and Section 7 draws the conclusions.

## 2. Satellites-Only and Performance Evaluation

Referring to Figure 1, having *N* different satellites in view from the lunar receiver, *N* pseudoranges can be measured. A common way to represent this situation is exploiting the following equation:

(1) $${\hat{\rho }}^{T}\left(\theta \right)=\left[\begin{array}{cccc} {\hat{\rho }}_{1}\left(\theta \right) & {\hat{\rho }}_{2}\left(\theta \right) & \ldots & {\hat{\rho }}_{N}\left(\theta \right) \end{array}\right]+n^{T}\left(\theta \right)$$

where the vector $\hat{\rho }$ is composed of the pseudorange measurements from each satellite, the state vector θ is composed of the problem unknowns (position and clock bias of the user), $\theta =(x_{u},y_{u},z_{u},dt_{u})$, and n(θ) is the measurement noise vector. Considering that the satellite *i* has a known position $s_{i}=\left(x_{i},y_{i},z_{i}\right)$, the i-th pseudorange has the following formulation:

(2) $$\begin{array}{c} \begin{array}{c} \rho_{i}\left(\theta \right)=\sqrt{{\left(x_{u}-x_{i}\right)}^{2}+{\left(y_{u}-y_{i}\right)}^{2}+{\left(z_{u}-z_{i}\right)}^{2}}+ct_{u} \end{array} \end{array}$$

and having N≥4 measurements, it is possible to linearize Equation (2), obtaining [24]:

(3) Δρ=HΔθ+n

where $\Delta \rho =\hat{\rho }\left(\theta \right)-\rho \left(\theta \right)$ is the difference between the measured and expected pseudoranges, $\Delta \theta =\hat{\theta }-\theta$ is the difference between the estimated and the real user state vector, and H is the Jacobian matrix of the vector ρ(θ).

It must be noted that this formulation is a common simplification of the localization problem in which the error contributions are all considered additive and independent, composing the vector n. Later, more details on the error model used in the paper will be given.

Exploiting the Weighted Least Square (WLS) approach, it is possible to estimate the user parameters:

(4) $$\Delta \theta ={\left(H^{T}N^{-1}H\right)}^{-1}H^{T}N^{-1}\Delta \rho$$

where N is the measurement error covariance matrix.

Two methods can be followed to evaluate the performance of the localization techniques: Cramér–Rao Lower Bound computation and system simulation, using Monte Carlo runs.

In the first case, assuming an unbiased estimator for the problem given in Equation (1), $\hat{\theta }\left(\rho \right)$, the Cramér–Rao inequality is given by the following expression [25]:

(5) $$C\left(\theta \right)=E\left\{\left(\hat{\theta }\left(\rho \right)-\theta \left(\rho \right)\right){\left(\hat{\theta }\left(\rho \right)-\theta \left(\rho \right)\right)}^{T}\right\}\geq {\left[J\left(\theta \right)\right]}^{-1}$$

Here, C(θ) represents the error covariance matrix related to the estimated vector of unknowns, while J(θ) refers to the Fisher Information Matrix (FIM) and its elements have the following form (in case of Gaussian distributed zero mean additive error):

(6) $$\begin{array}{cc} \; & J{\left(\theta \right)}_{m,k}=\frac{\partial \rho (\theta )}{\partial \theta_{m}}N{\left(\theta \right)}^{-1}\frac{\partial \rho (\theta )}{\partial \theta_{k}}+\\ \; & +\frac{1}{2}tr\left(N{\left(\theta \right)}^{-1}\frac{\partial N(\theta )}{\partial \theta_{m}}N{\left(\theta \right)}^{-1}\frac{\partial N(\theta )}{\partial \theta_{k}}\right) \end{array}$$

where ${\frac{\partial }{\partial \theta }}_{i}$ represents the derivative w.r.t. the i-th component of the parameter vector θ, $\frac{\partial \rho (\theta )}{\partial \theta }$ corresponds to the matrix H, and N(θ) is the covariance matrix of the measurement error n(θ) introduced in Equation (1).

The overall error associated with each pseudorange can be subdivided into distinct contributions, each independent of the others: space segment errors (satellite clock and position errors), propagation errors (atmospheric-related errors and multipath), and receiver measurement noise. In the context of Moon navigation, atmospheric effects on the signal propagation can be neglected, as the Moon can be considered to be surrounded by vacuum [26], and the total measurement error is given by four main contributions:

1. The satellite position error, which depends on the ephemeris error in the navigation message and it is assumed equal to 10 m (1σ) [27,28,29,30,31,32]. In particular, the most recent lunar satellite navigation-related study [31] can be considered as the source for the reference satellite position performance for this work, assuming the ephemeris validity period (and their renewal time) is smaller than 4 h.
2. The satellite clock ($n_{S-Ck}$) error, which results from clock time deviations and is assumed to be as proposed in [14,33] with Deep Space Network synchronization every 24 h, obtaining an error within 10 ns with respect to UTC [34].
3. The multipath error, where the open-sky Brenner’s multipath model is used to estimate the multipath error [35] with the following assumptions [14]: Binary Phase Shift Keying (BPSK) signals, rectangular spreading symbols with a 1 × 1.023 MHz spreading code rate, and a receiver front-end bandwidth of 8 MHz.
4. For the receiver error ($n_{RX}$), the same assumptions of [14] are used, obtaining a receiver error standard deviation of 0.95 m.

In the CRLB computation, if the satellite position is not directly added to the pseudorange, typically, the matrix N is considered diagonal, with the elements on the diagonal computed as follows, as in the case of computation of Dilution Of Precision (DOP) [24]:

(7) $$\sigma_{PR}^{2}=\sigma_{RX}^{2}+\sigma_{S-p}^{2}+\sigma_{M}^{2}+\sigma_{S-Ck}^{2}$$

where $\sigma_{RX}^{2},\sigma_{S-p}^{2},\sigma_{M}^{2},\sigma_{S-Ck}^{2}$ are the variances of the receiver measurement error, satellite position error, multipath error, and satellite clock error, respectively.

Having C(θ), it is possible to compute the standard deviation for the 3D horizontal and vertical position errors [24,36]:

(8) $$\begin{array}{cc} \; & \sigma_{3D}=\sqrt{C{\left(\theta \right)}_{11}+C{\left(\theta \right)}_{22}+C{\left(\theta \right)}_{33}}\\ \; & \sigma_{Hor.}=\sqrt{C{\left(\theta \right)}_{11}+C{\left(\theta \right)}_{22}}\\ \; & \sigma_{Vert.}=\sqrt{C{\left(\theta \right)}_{33}} \end{array}$$

Alternatively, as mentioned before, the performance can be evaluated by simulating the scenario and the user localization algorithm and evaluating the final error statistics using Monte Carlo runs. In detail, the simulation steps are:

1. Trajectory Simulation: Nominal satellite and user trajectories are generated.
2. Satellite Position Error: Gaussian, uncorrelated noise is added to each coordinate of the satellite trajectory.
3. Pseudorange Computation: the pseudorange is computed by also adding the RX clock bias and the other sources of errors (RX, multipath, and satellite clock all independent, uncorrelated, and Gaussian-distributed):
  (9) $$\begin{array}{c} \rho_{i}\left(\theta \right)=\sqrt{{\left(x_{u}-x_{i}-\epsilon_{x}\right)}^{2}+{\left(y_{u}-y_{i}-\epsilon_{y}\right)}^{2}+{\left(z_{u}-z_{i}-\epsilon_{z}\right)}^{2}}+ct_{u}+\\ +\epsilon_{M}\left(\theta \right)+\epsilon_{RX}+\epsilon_{S-Ck} \end{array}$$
  Each error term, ϵ, is modeled as a zero-mean Gaussian distribution, with a specified standard deviation, as described in the paper. Moreover, the standard deviation of the multipath error depends on the satellite’s elevation angle, as described in the paper.
4. Position Estimation: The iterative WLS algorithm is used to estimate the receiver position for each trajectory point.
5. Statistical Evaluation: For each point, *N* Monte Carlo trials are performed, and the root mean square (RMS) error across trials is computed.

In this way, the geometrical user-satellite effects are taken into account when introducing the satellite position error, together with a realistic time bias of the receiver. The other error contributions are merely added to the expected pseudorange.

Finally, the same approaches for CRLB computing or Monte Carlo simulation can also be used in case of additional measurements, as shown in the following sections.

## 3. Differential Reference Station

A local sensor based on the Moon can produce and broadcast differential corrections as in GNSS [24,36]. The local sensor can be installed on the lunar surface at the south pole region, i.e., the target area for future lunar exploration missions [1]. The station generates and transmits the pseudorange corrections based on the pseudorange measurements of all visible satellites. The station should be composed of a satellite navigation receiver and an antenna, used to compute the pseudorange and its position, and a communication system to send corrections to the user. Corrections can be sent directly to the user or via satellites, if the navigation system is combined with a satellite communication system, as often proposed [37,38], and exploited by the user to reduce its error component.

This configuration is the simplest and least expensive of the three proposed here, requiring only a data link receiver on the user side. The DRS generates the pseudorange corrections based on the pseudorange measurements from visible satellites:

(10) $$\delta \rho_{i}=\rho_{i}-R_{i,r}=ct_{r}+n_{i,r}\;\;\;i=1,\ldots ,N$$

where $R_{i,r}$ is the distance between the satellite and the reference stations (known), and $n_{i,r}$ is the additive measurement error. The correction is sent to the user and exploited to reduce the error component:

(11) $$\rho_{i}-\delta \rho_{i}=R_{i,u}+ct_{u}-ct_{r}+n_{i,u}-n_{i,u}\;\;\;i=1,\ldots ,N$$

On the Moon, the errors that can be reduced by differentiating the measurements are the satellite position error and the satellite clock error. Remembering that no important propagation errors (apart from multipath) are present and recalling that the satellite clock error is exactly the same for the reference and user, the final error contribution after the differential correction is as follows:

(12) $$n_{i,u}-n_{i,r}=\left(n_{i,u}^{S-p}-n_{i,r}^{S-p}\right)+\left(n_{i,u}^{RX}-n_{i,r}^{RX}\right)+\left(n_{i,u}^{M}-n_{i,r}^{M}\right)$$

where the terms in the last two couples (receiver and multipath errors, respectively) are uncorrelated but small and the first couple represents the residual error due to the satellite position error.

**Figure 2:**
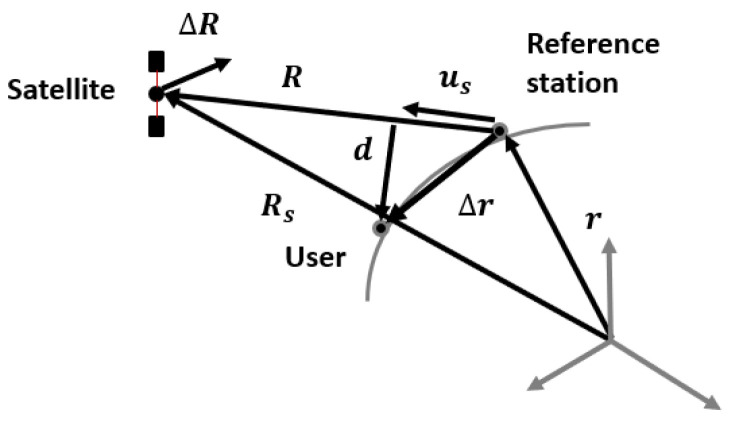
Vector relationship for the satellite position error computation in the differential approach.

Referring to Figure 2, the difference between satellite position errors ($n_{i,u}-n_{i,r}$) depends on the geometry and can be derived as in [23]: a satellite ephemeris error, ΔR, causes a scalar ranging error *E*, that is, the projection of the satellite vector error on the direction given by the unit vector pointing the satellite from the user, $u_{S}$:

(13) $$E=\Delta R^{T}\cdot u_{S}=\Delta R^{T}\cdot \frac{R}{R}$$

From Figure 2, $R=R_{S}-r$ and $u_{S}=\frac{R_{S}-r}{\left|\left|R_{S}-r\right|\right|}=\frac{R}{R}$, and the variation in error due to the geographical displacement between the user and the reference, ΔE, is given by:

(14) $$\Delta E≅\frac{\partial E}{\partial r}\cdot \Delta r=\frac{-\Delta R^{T}\cdot d}{R}$$

where $d=\left[\Delta r-\left(u_{S}^{T}\cdot \Delta r\right)u_{S}\right]$. Finally, noting that d<Δr, the residual error absolute value is limited by:

(15) $$\left|\Delta E\right|\leq \frac{\left|\Delta r|\cdot |\Delta R\right|}{R}$$

where |Δr| is the user–local sensor distance and *R* is the satellite distance.

Equation (15) indicates that the magnitude of the satellite position error, |ΔR|, is reduced by a factor of $\frac{\left|\Delta r\right|}{R}$ and the variance of the pseudorange error, after differentiating, becomes:

(16) $$\sigma_{diff}^{2}={\left(\frac{\left|\Delta R\right|}{R}\sigma_{S-p}\right)}^{2}+2\cdot \sigma_{RX}^{2}+2\cdot \sigma_{M}^{2}$$

This last equation allows for the computation of the error budget for the CRLB computation in case of DRS, by substituting Equation (7) in the formulation of the problem.

Also, for the DRS, the performance can be estimated using Monte Carlo trials, with the following main steps:

- Trajectory and Sensor Simulation: nominal satellite, user, and local sensor positions are generated.
- Error Injection: Gaussian, uncorrelated noise is added to both the satellite and local sensor positions.
- Pseudorange Generation: user and sensor pseudoranges are computed with the inclusion of receiver clock bias and other error sources (receiver noise, multipath, satellite clock), all modeled as independent Gaussian noise.
- Differential Correction: the differential corrections are computed and applied to the user pseudoranges.
- Position Estimation and Analysis: the iterative WLS algorithm is used for position estimation at each trajectory point, and *N* trials are conducted per point to evaluate the RMS error.

Finally, it must be noted that on the Moon, the ionospheric and tropospheric effects are negligible, and this approach can have higher performance than on Earth. In fact, neglecting the ionospheric and tropospheric errors, the most significant error contributions are given by the satellite errors (position and time) that are usually much more correlated in space and time, resulting in higher accuracy and higher coverage at the end of the process.

## 4. Pseudolite

Pseudolites are ground-based transmitters that broadcast signals similar to satellites to improve navigation performance in areas where satellite signals are weak or unavailable. The pseudolite should be composed of a satellite navigation receiver and antenna to compute its own position and to be synchronized with the satellites, and an omnidirectional transmitter, in the same band, able to generate a ranging signal similar to the one produced by satellites. Like the previous one, this solution is simple and cost-effective and does not need any modifications on the user side: the user will track the pseudolite signal together with the satellite signals, exploiting the same receiver. As in the case of terrestrial pseudolite, the near–far problem must be taken into account for this configuration [39,40], and also the coverage of the local sensor is reduced, due to visibility and link budget constraints.

The additional measurement from the pseudolite has the same formulation as the pseudorange and can be directly added to the system of equations introduced in Equation (3):

(17) $$H=\left[\begin{array}{c} H_{PR}\\ H_{PL} \end{array}\right];\;\;n\left(\theta \right)=\left[\begin{array}{c} n_{PR}\left(\theta \right)\\ n_{PL}\left(\theta \right) \end{array}\right]$$

where $H_{PR}$ is the matrix corresponding to the satellite pseudorange measurements, $H_{PL}$ is the matrix (vector in case of one pseudolite) corresponding to the pseudolite pseudorange measurement, $n_{PR}\left(\theta \right)$ is the pseudorange measurement error vector, and $n_{PL}\left(\theta \right)$ is the pseudolite measurement error vector. The measurement errors to be taken into account for the pseudolite are similar to those described earlier for the satellite, but additional considerations arise due to the relatively close proximity of the receivers to the pseudolite. The following assumptions for the mentioned error sources were made: the pseudolite position error is modeled as Gaussian distributed, with zero mean and a standard deviation of 0.1 m ($\sigma_{LE-pos}$); the pseudolite is considered synchronized with the satellite constellation and its clock error is modeled as previously described in Section 2; for the Multipath error, the same model as before (open-sky Brenner model) is used, with a standard deviation of one meter.

## 5. Independent Source of Localization

A new type of local sensor is proposed, which supplies signals for three additional measurements: elevation, azimuth, and range, allowing, when the number of navigation satellites is too low, a complete autonomous 3D positioning or joint position estimation with the satellites in view.

This configuration is the most expensive and complex, it comprises a satellite navigation receiver and an independent localization system receiver, for the user, and the local sensor is composed of four transmitters/receivers equipped with phased array sector antennas (e.g., with an azimuth coverage angle of 90 degrees and an elevation coverage angle of 60 degrees each) to cover all the possible azimuth angles. Each sector antenna must allow measurement of the horizontal and vertical angles of arrival of the incoming signals by exploiting digital beam-forming techniques.

Additionally, the use of two-way communication enables the measurement of the distance between the spacecraft/rover and the local sensor.

The working principle of the ISL can be outlined in the following steps: the user interrogates the local sensor using an omnidirectional antenna, the local sensor measures the angle of arrival of the interrogation and sends back a reply containing the measured angle with a fixed time of reply, the user computes the range exploiting the travel time and reads the angle of arrival on the reply, and the user computes its position exploiting the three measurements and the measurement from satellites, if available.

As mentioned before, the user must be equipped with a specific transponder to communicate with the local sensor and the coverage area is limited due to visibility and link budget.

The elevation and azimuth measurements in East North Up (ENU) coordinates centered on the local sensor are given by the following expression:

(18) $$\hat{El}\left(\theta_{ENU}\right)=El\left(\theta_{ENU}\right)+n_{El}=\tan^{-1}\left(\frac{z^{ENU}}{R_{H}}\right)+n_{El}$$

(19) $$\hat{Az}\left(\theta_{ENU}\right)=Az\left(\theta_{ENU}\right)+n_{Az}=\tan^{-1}\left(\frac{y^{ENU}}{x^{ENU}}\right)+n_{Az}$$

where $R_{H}=\sqrt{{\left(x^{ENU}\right)}^{2}+{\left(y^{ENU}\right)}^{2}}$ is the horizontal distance between the user and the local sensor. These measurements must be expressed in Lunar-Centered Lunar-Fixed (LCLF) coordinates to solve the general localization problem, exploiting the following transformation matrix:

(20) $$\begin{array}{cc} \; & X^{ENU}=G\left(X-X_{0}\right)\\ \; & G=\left[\begin{array}{ccc} -\sin\gamma & \cos\gamma & 0\\ -\cos\gamma \sin\varphi & -\sin\gamma \sin\varphi & \cos\varphi \\ \cos\gamma \cos\varphi & \sin\gamma \cos\varphi & \sin\varphi \end{array}\right] \end{array}$$

where $X^{ENU}={\left(x^{ENU},y^{ENU},z^{ENU}\right)}^{T}$ is the 3D coordinates vector of the user in ENU coordinates, $X_{0}=\left(x_{LE},y_{LE},z_{LE}\right)$ is the local sensor position in LCLF coordinates, X is the position vector of the user in LCLF, and γ and φ are the longitude and latitude, respectively, of the local sensor.

Again, the H matrix and the covariance matrix of the measurement can be used to compute the system performance. The H matrix can be computed as the Jacobian of the composite function, obtaining:

(21) $$H_{Az/El}=H_{Az/El}^{ENU}\cdot G$$

where $H_{Az}^{ENU}=\left[\frac{-y^{ENU}}{R_{H}^{2}},\frac{x^{ENU}}{R_{H}^{2}},0\right]$ and $H_{El}^{ENU}=\left[\frac{-x^{ENU}\cdot z^{ENU}}{R_{3D}^{2}\cdot R_{H}},\frac{-y^{ENU}\cdot z^{ENU}}{R_{3D}^{2}\cdot R_{H}},\frac{R_{H}}{R_{3D}^{2}}\right]$.

The range measurement can be formulated as follows:

(22) $$R\left(\theta \right)=\sqrt{{\left(x-x_{\mathrm{LE}}\right)}^{2}+{\left(y-y_{\mathrm{LE}}\right)}^{2}+{\left(z-z_{\mathrm{LE}}\right)}^{2}}+n_{R}$$

obtaining, for the H matrix:

(23) $$H_{R}=\left[\begin{array}{ccc} \frac{x-x_{LE}}{R_{3D}} & \frac{y-y_{LE}}{R_{3D}} & \frac{z-z_{LE}}{R_{3D}} \end{array}\right]$$

where $R_{3D}=\sqrt{{\left(x-x_{LE}\right)}^{2}+{\left(y-y_{LE}\right)}^{2}+{\left(z-z_{LE}\right)}^{2}}$.

The final H matrix is composed of the three matrices relative to satellite measurements, sensor range, and angle measurements (zero padding is applied for compatibility):

(24) $$H=\left[\begin{array}{c} H_{PR}\\ H_{R},0\\ H_{EL},0\\ H_{AZ},0 \end{array}\right];\;\;n\left(\theta \right)=\left[\begin{array}{c} n_{PR}\left(\theta \right)\\ n_{R}\left(\theta \right)\\ n_{El}\left(\theta \right)\\ n_{Az}\left(\theta \right) \end{array}\right]$$

As before, to evaluate the user accuracy, it is also necessary to derive the measurement error model for $n_{R}$ and $n_{AZ/EL}$. The range measurement error due to the receiver noise can be assumed as Gaussian distributed, zero mean, and standard deviation $\sigma_{R}$ derived from the literature [41]:

(25) $$\sigma_{R}=\frac{c}{2\cdot \mathrm{BW}\cdot \sqrt{N\cdot SNR}}$$

where *c* is the speed of light, BW is the signal bandwidth, *N* is the integration factor (usually equal to the number of available measurements, for example, using more pulses or different snapshots of the incoming signal), and SNR is the signal-to-noise ratio.

Angle measurement errors can be assumed composed of two different contributions: (1) a measurement error, with the assumption of Gaussian distribution, zero mean, and standard deviation $\sigma_{AOA}$, taking into account the effect of the SNR on the measurement process in the receiver and (2) the error due to the station position uncertainty and/or the unknown user antenna–body displacement. The first contribution, $\sigma_{AOA}$, can be computed recalling the antenna array parameters, with the following formula [41]:

(26) $$\sigma_{AOA}=\frac{\theta_{3}}{k_{m}\cdot \sqrt{2\cdot N\cdot SNR}}$$

where $\theta_{3}$ is the half-power beam width (for a generic aperture antenna, $\theta_{3}=70*\frac{\lambda }{D}$), $k_{m}$ is a multiplicative factor that takes into account the antenna beam shape, *N* is the integration factor, and SNR is the signal-to-noise ratio. Finally, λ is the wavelength and *D* is the antenna dimension [42].

In the case of a super-resolution processor, Equation (26) becomes, assuming the use of the MUSIC algorithm [43]:

(27) $$\sigma_{AOA}=\sqrt{\frac{6}{m^{3}\cdot N\cdot SNR}}$$

where *m* is the number of elements of the antenna ($m=\frac{2D}{\lambda })$ and *N* is the integration factor.

In the following, *N* will be assumed equal to the number of bits of the preamble of the message discussed later on.

The error due to the station position error can be derived from the propagation of uncertainty theory [44]. Recalling that the usual assumption is that the reference station position is previously calculated with a long-term observation or with independent localization systems such as the Laser Retroreflector Array (LRA) for Lunar Laser Ranging and/or X-band transponder for Range and Range Rate (RARR), and Delta Differential One-way Ranging (DDOR) exploiting Earth tracking stations, it is possible to assume that the station position error is very small, independent, and uncorrelated to the other sources.

Assuming also that the local sensor errors on x,y,z have the same standard deviation σ, and recalling that the derivatives of the elevation and azimuth functions with respect to (x,y,z) correspond to the elements of the Jacobian matrix $H_{Az/El}^{ENU}$, the elevation and azimuth error variances are given by the following equations:

(28) $$\begin{array}{cc} \sigma_{El}^{2}=\; & \sigma^{2}\left(\frac{R_{H}^{2}\cdot R_{3D}^{2}}{R_{3D}^{4}\cdot R_{H}^{2}}\right)=\frac{\sigma^{2}}{R_{3D}^{2}} \end{array}$$

(29) $$\begin{array}{cc} \; & \sigma_{Az}^{2}=\sigma^{2}\left(\frac{{\left(y-y_{LE}\right)}^{2}}{R_{H}^{4}}+\frac{{\left(x-x_{LE}\right)}^{2}}{R_{H}^{4}}\right)=\frac{\sigma^{2}}{R_{H}^{2}} \end{array}$$

Finally, to compute the total angle error standard deviation, the two error contributions must be summed:

(30) $$\sigma_{El/Az}=\sqrt{\sigma_{AOA,El/Az}^{2}+\sigma_{El/Az}^{2}}$$

Note that the first contribution depends on the SNR, is proportional to ($R_{3D}^{2}$), and is predominant for large distances, while the second contribution is proportional to $R_{3D}^{-2}$ and $R_{H}^{-2}$ for elevation and azimuth measurements and is predominant for short distances ($R_{H}$ can be also replaced by $R_{3D}$, having a small overestimation of the error).

Last but not least, recalling Equations (25) and (26), it is clear that the measurement performance also depends on other important parameters, such as the integration factor, *N*, the instantaneous band of the signal, BW, the antenna dimension, *D*, and the carrier wavelength, λ. For this reason, a preliminary design of the signals and protocols of the local sensor is proposed.

Recalling Equation (26), higher frequencies guarantee higher performance with a small antenna. Moreover, recalling that the Interagency Operations Advisory Group (IOAG) has defined a series of standards and protocols for the lunar communication and navigation architecture [45], the Ka-band is here proposed to allow the use of a small antenna for simple integration on the ISL (D=0.5 m is considered hereafter).

The maximum coverage of the local sensor was fixed to 20 km, assuming a minimum SNR of 10 dB at 20 km. BPSK modulation was selected for robustness and reliability, obtaining a Bit Error Rate (BER) of $10^{-3}$ at 20 km [42]. Finally, the BW was set to 2 MHz.

A random access to the channel was chosen due to its simplicity, no need of channel management, the expected low number of simultaneous users, and throughput. Moreover, random access simplifies the time of flight measurement process, as in other navigation systems, such as the Distance Measurement Equipment (DME) [46].

The proposed realistic assumptions allow the selection of the parameters *D*, SNR, and λ to compute the needed standard deviation of the AOA. To obtain realistic values for *N*, knowing the modulation scheme and the signal band, it is necessary to make additional assumptions about the duration of the message time and the number of bits.

Three types of messages are proposed. A Local Element Initialization message (LEI) is broadcast periodically by the local sensor to let the user know that it is in coverage of the local sensor and to obtain the local sensor information, i.e., the sensor ID and its position. A User Interrogation message (UI) contains the user and the local sensor IDs, used to start the measurement process. It is periodically repeated during navigation. For a Local Element Reply message (LER), for each received UI, the local sensor computes the angles of arrival (elevation and azimuth), and the message replays after a fixed delay. The message contains the measured angles, the beacon station ID, and the user’s ID. Upon receiving the LER, the user can compute the round-trip delay (i.e., the range) from the local sensor and decode the AOA information encoded on it.

**Figure 3:**
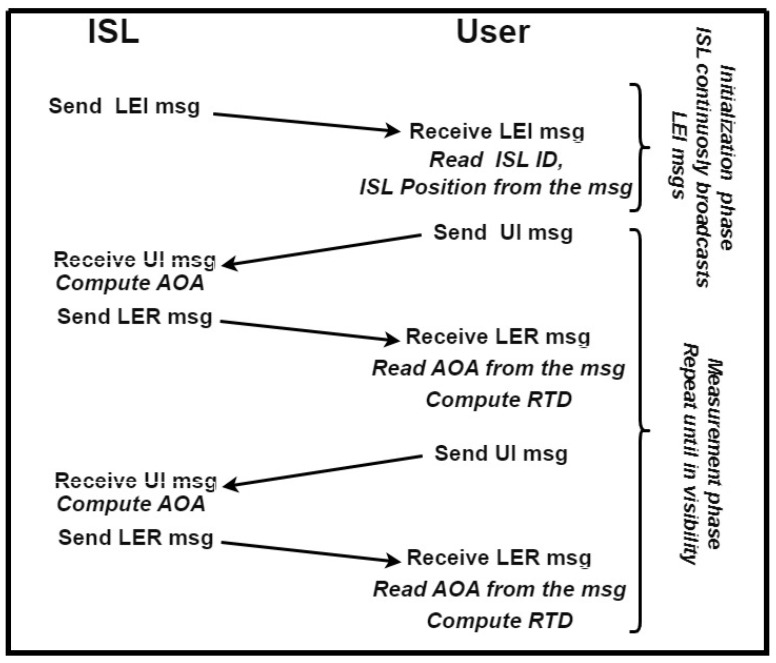
ISL working principle and data communication chart.

The working principle, data, and message flow of the proposed method are reported in Figure 3.

The messages include a preamble, a data block, and a parity check field. The data block includes the message type that defines the data content.

Concerning the dimensions of each field, they were chosen similar to the dimensions of analogous fields used in other long-time tested protocols such as the ADS-B protocol [47,48]. The preamble is used to synchronize the reception and to perform the measurements; its dimension will be discussed later. The “type” field is 8 bits long and is used to specify what type of message is being sent and/or different submessage types. A parity check field that keeps the checksum value of the entire message is 16 bits long.

The data field will be different for different types of messages: UI and LER messages contain the user and the local sensor IDs, which are assumed to be 16 bits long. The LER message also contains the measured angle information: 16 bits for the elevation and 16 bits for the azimuth, as well as 16 bits for the ID station. The LEI message comprises the ID of the local sensor and its position information, i.e., latitude, longitude, and altitude. The latitude and longitude are 32 bits and the altitude is 24 bits.

Note that this is only a possible implementation, subject to improvement and changes, but, for the scope of the paper, it is enough to have a first assessment of channel occupancy, AOA, and range measurement accuracy.

Under these conditions, the maximum number of data bits in each message, without considering the preamble, is 128 bits, and the length of the preamble can be chosen as long as possible to increase the accuracy of the angle and range measurement. In fact, the preamble bits will be used to make *N* different measurements on the same messages, obtaining *N* as the integration factor.

To set a value for the preamble size in terms of number of bits, which corresponds to the integration factor *N*, the message time duration must be selected: the message should be as long as possible to improve the AOA measurement performance, but it also has a constraint due to the channel occupancy. A derivation to set a value of the message’s time length (in terms of number of users and probability of successful localization) follows.

Assuming we have $N_{user}$ users transmitting interrogations, each with a rate *r*, that the message is $t_{int}$ seconds long, and that to assure the service it is enough to take at least one measurement over *M* (e.g., at least one measurement over 5 s with an interrogation rate of 1-second means one measurement over 5, M=5), the following formula gives the successful transmission probability:

(31) $$P_{succ}=1-{\left(1-Pr\left(0\right)\right)}^{M};\;Pr\left(0\right)=e^{\left(-2\lambda t_{int}\right)}$$

where $\lambda =N_{user}\cdot r$, and the expected probability of no collision, Pr(0), is approximated by the Poisson distribution [49]. Note that (1−Pr(0)) is the probability that a collision occurs for one message, ${\left(1-Pr\left(0\right)\right)}^{M}$ is the probability that collisions occur for *M* messages, and $P_{succ}=1-{\left(1-Pr\left(0\right)\right)}^{M}$ is the probability of having at least a message without collision on *M* received messages.

Fixing the number of simultaneous users $N_{user}$ to 10, the signal time duration of 1 ms, the packet transmission rate, *r*, to 2 packets per second, and requiring at least one measurement every one second, the probability of successful transmission for the user is 0.9985.

Recalling that a 2 MHz bandwidth BPSK modulation was proposed, a bit−rate of 1 Mbit/s is obtained, i.e., the total number of bits for a single message can be slightly higher than 1000 (accepting $P_{succ}$ on the order of 0.99).

Summing up, a message with a time-size in the order of one millisecond allows high performance in terms of probability of correct transmission and allows a preamble bit-size in the order of one thousand (i.e., N=1000).

In Table 1, a summary of the assumptions is reported: with a preamble of ca. 1000 bits, an integration factor of 1000 is obtained (considering one measurement for each bit of the preamble), $k_{m}$ was posed equal to 1.6 (according to [41]), obtaining $\sigma_{AOA}=0.0077$ degrees or, according to Equation (27), $\sigma_{AOA}=0.002$ degrees in case of MUSIC angle estimation and a range error standard deviation below one meter.

As mentioned above, with respect to angle error, for close distances, the predominant error is due to the glint error, which reaches values of almost up to 0.06 degrees at 100 m from the station, but it becomes small as the distance from the beacon increases. For large distances, the predominant error comes from the limit of the angle measurement noise, which at 20 km reaches 0.0077 degrees. Finally, by imposing a minimum distance from the local sensor of 600 m, the total angle error can be bounded to a maximum of 0.01 degrees for every possible distance (between 600 m and 20 km). Hereafter, for simulation, a constant value of 0.01 degrees will be assumed and an additional margin to take into account other secondary error contributions such as multipath effect on the angle estimation, errors due to digitalization, etc., will be considered. The same approach is used for the measurement error of the range where secondary effects, such as multipath, station position error, and transponder jitters, are taken into account doubling the receiver range measurement error, obtaining $\sigma_{R}=1.66$ m.

## 6. Evaluation on a Possible Lunar Constellation

The baseline for the performance evaluation is the satellite navigation system proposed in [13,14], exploiting pseudorange measurements from satellites in ELFO orbits, with a constellation of four satellites.

A detailed description of the constellation is out of the scope of this work and has already been performed by the authors in [13,14,15,50,51], who showed that the constellation parameters are as follows: # of satellites and planes = 4, Semi-major axis (a) and Eccentricity (e) equal to 9750 km and 0.6–0.7 respectively, Inclination (i) and Orbital Period (T) equal to 48–57 deg and 24 h, respectively.

The user scenario consists of a spacecraft following a linear descending trajectory, starting at $(-89.34^{\circ },90^{\circ },20\;$ km) and ending at $(-89.98^{\circ },90^{\circ },0\;$ m) and the local sensor located at the south pole of the Moon at 10 m of altitude, coordinates $(-90^{\circ },0^{\circ },10\;$ m). The local sensor is considered usable for independent measurement (pseudorange, range, and angles) if the user is closer than 20 km; therefore, only satellite measurements can be exploited.

The descending phase takes 100 min (well below the ephemeris validity period), and to evaluate the performance in different constellation conditions, three landing phases starting at three different constellation epochs were evaluated: the first starts at epoch 1350, the second starts at epoch 1600, and the third starts at epoch 2100. The timing of the three approaches was chosen so that it is possible to evaluate the proposed methods in three different constellation conditions: landing #1 has 4 satellites in view at all times, landing #2 has fewer than 3 satellites in view (not allowing localization with only satellites and satellites plus pseudolite configuration), and landing #3 has three satellites in view at all times.

The CRLB and r.m.s. error (r.m.s error is computed on each epoch of the scenario exploiting 200 Monte Carlo runs of a Weighted Least Square positioning algorithm) were evaluated for each landing trajectory and compared in the following figures, one for each landing trajectory. Each figure reports both the vertical and the horizontal error to provide a clearer understanding of the error trends.

In each figure, the vertical gray line shows the moment at which the PSL and the ISL start to be in view from the user, and the error is reported in log-scale to better distinguish the difference among the various methods. Finally, the local sensor configurations are as follows: only satellites (No local sensor), satellites plus pseudolite (PSL), satellites plus independent source of localization (ISL), satellites plus differential correction (DRS), satellites plus differential correction plus pseudolite (DRS+PSL), and satellites plus differential correction plus independent source of localization (DRS+ISL).

**Figure 4:**
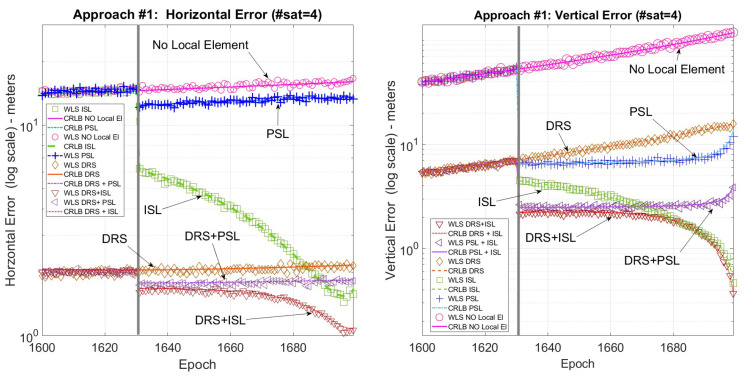
Landing #1. CRLB/WLS horizontal and vertical accuracy for the descending trajectory.

**Figure 5:**
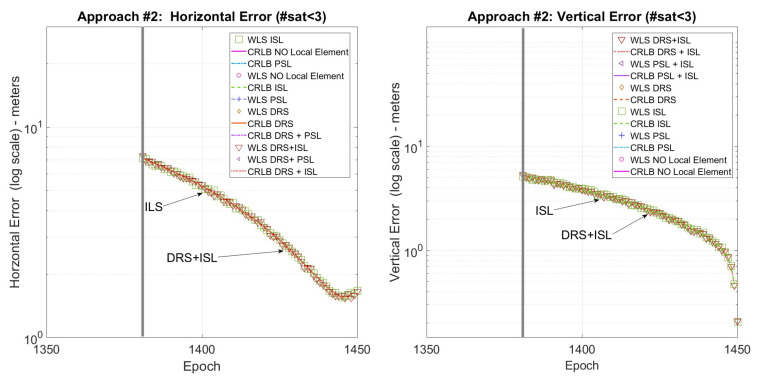
Landing #2. CRLB/WLS horizontal and vertical accuracy for the descending trajectory.

**Figure 6:**
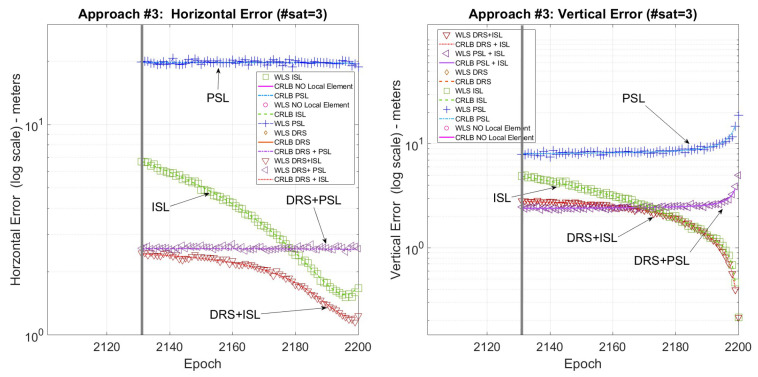
Landing #3. CRLB/WLS horizontal and vertical accuracy for the descending trajectory.

Figure 4 shows the horizontal and vertical error for the first landing trajectory where all the techniques are available. Figure 5 shows the horizontal and vertical error for the second landing trajectory where, due to the lack of satellites, only the ISL is available for positioning. Finally, Figure 6 shows the horizontal and vertical error for the third landing trajectory where, using a pseudolite, it is possible to use the three satellites in view for positioning.

Focusing on Figure 4, it is possible to note that the positioning error is greatly reduced when using the local sensor, especially the vertical one, obtaining the best performance when using DRS+ISL. Furthermore, also when the local sensor is used only for differential corrections there is a great improvement especially for the horizontal error, where the DRS performs even better than (or equal to) the other methods.

Summarizing, DRS+ISL always has the best performance: both the horizontal and vertical errors are about 2 m in the initial part of the trajectory and in the last part around 1 m for the horizontal error and well below 1 m for the vertical error.

Figure 5 and Figure 6 show that when fewer than four satellites are visible, exploiting the ISL or the PLS solutions, it is still possible to solve the positioning problem. Moreover, ISL+DRS gives the best results, similar to the previous case, also when only one satellite is in visibility.

Finally, to have a term of comparison with possible navigation requirements, Table 2 reports the comparison of the obtained performances with the requirements proposed by ESA [52] for descent approach and terminal landing phases of a lunar mission. This comparison is performed for the three landing scenarios in terms of the percentage of epochs for which the requirements are satisfied.

During the descent phase, from 28 km of altitude down to about 3 km, the horizontal requirement is satisfied with any method (if available) and the availability of the service improves from 33% to 77% with the ISL solution. Concerning vertical error, more complex solutions, such as ISL + DRS, are essential to increase availability from 0% to 56%.

In the ESA Terminal Landing phase, from about 3 km of altitude to the lunar surface, the requirements are quite stringent: the vertical error requirement is never satisfied, and the horizontal requirement can be satisfied only if the local sensor is exploited (ISL + DRS). This result calls for additional investigations on autonomous techniques for vertical guidance, such as laser altimeters, cameras, etc., that should be conducted on board for the landing phase.

**Figure 7:**
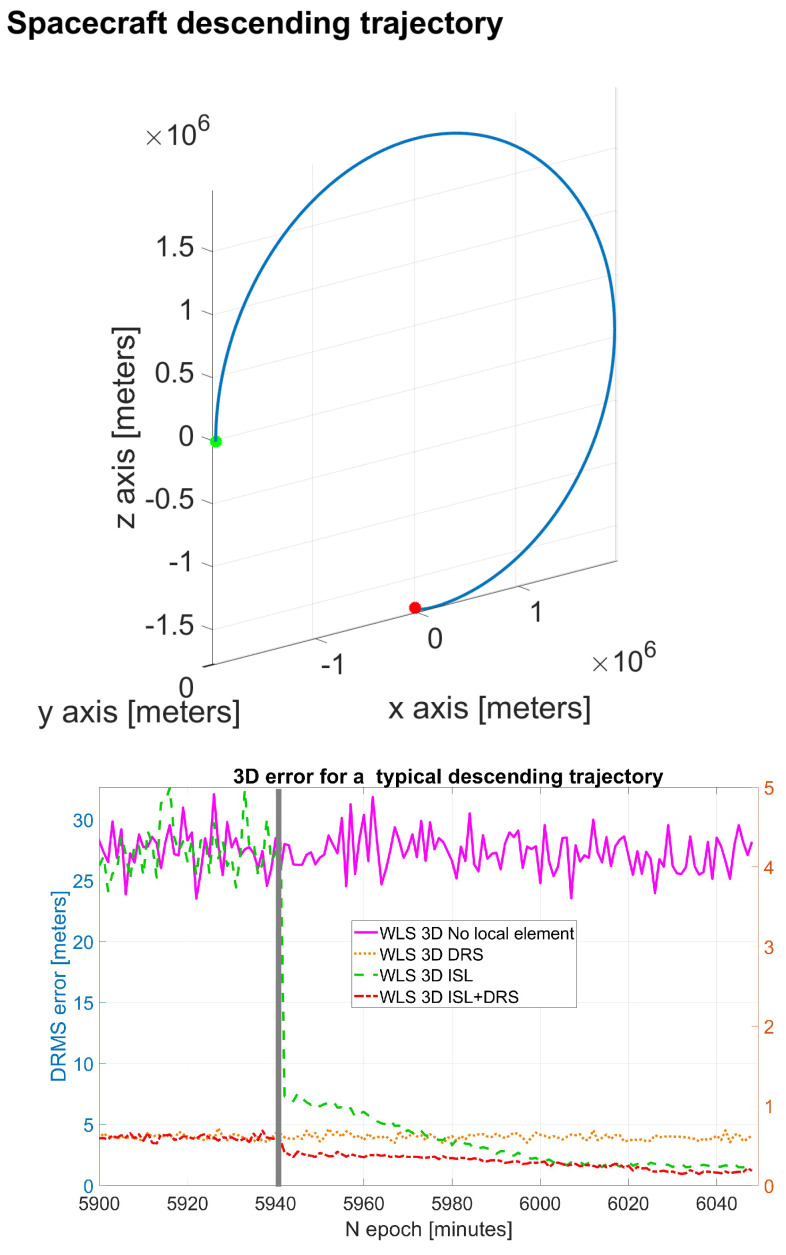
Landing #4. RMS 3D error for a descending trajectory from a low altitude transfer orbit to the south pole. Up: trajectory, Down: 3D RMS errors.

Previous results are also confirmed from other scenarios, such as the one reported in the following example, where a spacecraft moves from a low orbit parking position to the Moon surface following a typical descending and landing trajectory (Figure 7—top).

The 3D RMS errors (Figure 7—bottom) of the most promising configurations (DRS, ISL, and ISL+DRS) confirm the previous simulation results, with DRS + ISL obtaining the best performance.

## 7. Discussion and Conclusions

Different solutions for a lunar local sensor were evaluated and compared. A new working principle was described for a local sensor (the ISL solution) and designed, proposing an initial version for the communication protocol.

The simulation results show that there is an increase in navigation accuracy using a local sensor, with a significant improvement when the proposed ISL solution is exploited.

Table 3 summarizes some final considerations. The proposed ISL performs as well as or even better than the DRS, providing a huge accuracy improvement for the vertical positioning error. For the horizontal error, the DRS performance can be further improved with the combined use of DRS+ISL. In terms of accuracy, in any case, the DRS+ISL configuration always provides the best performance.

It is also important to consider that the DRS solution does not have important limitations in terms of coverage [23], and that the error reduction is huge even for a baseline of hundreds of kilometers. At the same time, the DRS does not improve the availability of the service. On the other hand, the ISL always enables the navigation service even without satellites in view. The table also reports the expected complexity and dimensions for the local sensor and the user device, considering the working principle and comments given in the previous sections.

In particular, it must be recalled that the introduction of the ISL calls for additional room on the local sensor for the phased array antennas and additional complexity due to the need for AOA estimation. Moreover, the user side also requires a more complex device to interrogate the local sensor. This increases cost and can reduce the initial reliability compared to a more standard solution such as the DRS.

It must be noted that other important parameters, such as power consumption, temperature management, lifetime, and enviromental factors, such as lunar dust on the antennas and signal propagation, are not considered in this study and will likely be analyzed in future works. Moreover, the authors would like to emphasize that such challenges are likely common across all systems involved in the Artemis exploration era, which envisions a sustained human presence on the Moon, and it is expected that these issues will be addressed through dedicated studies beyond the scope of this work.

Finally, although in the authors’ opinion the proposed models are sufficient to provide an initial and reasonably accurate estimation of the expected performance, the introduction of time-correlated errors and non-Gaussian distributed models for the measurement errors could further enhance the statistical and mathematical frameworks used for system evaluation, leading to a more precise performance estimation.

In conclusion, in the opinion of the authors, the exploitation of differential corrections can be an effective initial solution, delivering significant improvements with minimal effort and cost, but a second step, the addition of ISL functionalities will be mandatory to increase accuracy, availability, and continuity of the service, to meet the stringent requirements of Moon missions and ensure a safe, precise lunar navigation and landing. Furthermore, multiple local elements could also be considered for more demanding scenarios in the future.

## Figures and Tables

**Table 1:** Local sensor parameters and errors assumption in the ISL configuration.

Parameter	Value
Max number of user	10
Min/Max range	600 m/20 km
Minimum SNR	10 db
Antenna hor/vert dimension	0.5 m
Signal Band	Ka
Signal Bandwidth	2 MHz
Modulation Type	BPSK
BER	$10^{-3}$
Probability of lack of service	0.99
Message length (number of bits)	1 ms (ca. 1000 bits)
N	1000
$\sigma_{LE-pos}$	0.1 m for each coordinate
$\sigma_{R}$	0.83 (+0.83) meters
$\sigma_{AOA}$	0.01 (+0.01) degree

**Table 2:** Percentages of time (epochs) that the ESA requirements are met. DA = Descent Approach, TL = Terminal Landing.

Performance on the 3 Trajectories				
**(% of Epochs Where the Error is Lower than the Requirement)**				
	**Normal**	**DRS**	**PSL [+DRS]**	**ISL [+DRS]**
DA HOR: 35 m	33%	33%	56% [56%]	77%[77%]
DA VER: 3.3 m	0%	0%	0% [44%]	36% [56%]
TL HOR: 2.3 m	0%	33%	0% [33%]	100% [100%]
TL VER: 0.16 m	0%	0%	0% [0%]	0% [0%]

**Table 3:** General performance comparison for the different configurations. - = no change, * = Very Low, ** =Low, *** = Medium, **** = High.

	DRS	PSL [+DRS]	ISL [+DRS]
Sensor Coverage	UP to 1000 km	Kilometers	Kilometers
Service Availability	-	**	***
Accuracy Improvement	****	*** [****]	**** [****]
User equip. complexity	**	- [**]	**** [****]
Sensor complexity	*	** [**]	*** [***]
Sensor weight and Dim.	*	[*]	*** [***]
Sensor cost	*	** [**]	**** [****]

## Data Availability

Data are contained within the article.
